# Draft Genome Sequence of the Trypanosoma cruzi B. M. López Strain (TcIa), Isolated from a Colombian Patient

**DOI:** 10.1128/MRA.00031-20

**Published:** 2020-04-30

**Authors:** Inmaculada Gómez, Alberto Rastrojo, Francisco José Sanchez-Luque, Fabián Lorenzo-Díaz, Francisco Macías, Basilio Valladares, Begoña Aguado, José María Requena, Manuel Carlos López, M. Carmen Thomas

**Affiliations:** a Instituto de Parasitología y Biomedicina López-Neyra, Consejo Superior de Investigaciones Científicas, PTS-Granada, Granada, Spain; b Centro de Biología Molecular Severo-Ochoa, Consejo Superior de Investigaciones Científicas, Madrid, Spain; c Instituto Universitario de Enfermedades Tropicales y Salud Pública de Canarias, Universidad de La Laguna, La Laguna, Spain; Vanderbilt University

## Abstract

Trypanosoma cruzi parasite strains are classified into six lineages (discrete typing units TcI to TcVI). The broad genetic diversity of T. cruzi strains has an influence on the development of the host response and pathogenesis, as well as drug susceptibility. Here, the draft genome of the T. cruzi B. M. López strain (TcIa) is reported.

## ANNOUNCEMENT

*Trypanosoma cruzi* causes Chagas disease, a chronic infection that affects around 8 million people worldwide and is a major social and public health problem in Latin America and Europe due to migratory movements (1, 2). Based on the genetic variability of T. cruzi, strains of this species are grouped into six discrete typing units (DTUs), TcI to TcVI (3). TcI, the most frequent and widely distributed T. cruzi DTU, is related to sylvatic and domestic environments, exhibits high genetic heterogeneity, and is associated with chagasic cardiomyopathy (4). Here, the draft genome of T. cruzi strain B. M. López (MHOM/CO/87), which was isolated from a patient from Paratebueno, Cundinamarca, Colombia, is reported (5). Lineage characterization (TcIa) was based on PCR amplification of the miniexon and nucleotide polymorphisms of the miniexon intergenic region (GenBank accession number MT231530) (6, 7).

Epimastigote forms of the parasite were grown at 28°C in liver infusion tryptose medium containing 10% heat-inactivated fetal bovine serum, and genomic DNA (gDNA) was purified by phenol-chloroform extraction and ethanol precipitation. Whole-genome sequencing was performed using Ion Torrent technology (Thermo Fisher). One microgram of gDNA was used for automatic library construction in the AB Library Builder system using the Ion Xpress Plus fragment library kit (Thermo Fisher); library size selection (approximately 480 bp) was performed using the E-Gel system and SizeSelect 2% agarose gels (Thermo Fisher). Library size was confirmed with a Bioanalyzer 2100 system using a high-sensitivity DNA kit (Agilent Technologies), and the DNA concentration was determined with the Quant-iT double-stranded DNA (dsDNA) assay kit and a Qubit fluorometer (Invitrogen). The gDNA library was diluted to 23 pM and subjected to emulsion PCR using the Ion OneTouch 400 template kit (Life Technologies). After enrichment, the final library was loaded on an Ion 316 v2 chip and sequenced using the Ion Torrent PGM platform with Hi-Q sequencing chemistry. A total of 5,415,819 raw reads, with an average size of 249 bp, were obtained and analyzed with FastQC v0.10.1 (www.bioinformatics.babraham.ac.uk/projects/fastqc) using default settings. Prinseq v0.20.4 (8) was used iteratively for quality filtering using the following parameters: derep, 14; ns_max_p, 1; ns_max_n, 3; trim_ns_left, 1; trim_ns_right, 1; trim_qual_right, 20; trim_qual_type, mean; trim_qual_window, 5; trim_qual_step, 1; trim_qual_right, 20; trim_qual_type, mean; trim_qual_window, 1; trim_qual_step, 1; trim_qual_left, 20; trim_qual_type, mean; trim_qual_window, 5; trim_qual_step, 1; trim_qual_left, 20; trim_qual_type, mean; trim_qual_window, 1; trim_qual_step, 1; lc_method, entropy; lc_threshold, 50; min_qual_mean, 25; and min_len, 50. With these parameters, 4,591,877 quality-filtered reads (average length, 254 bp) were obtained.

The genome was assembled, using CLC Genomics Workbench v8.0 (Qiagen) (length fraction, 0.90; similarity fraction, 0.97; minimum contig length, 500 bp), into 5,923 contigs totaling 18,508,455 bp, with an *N*_50_ value of 5,125 bp and an average contig size of 3,124 bp. The longest contig was 45,876 bp, and the genome G+C content was 48.26%. BUSCO v4.0.5 analysis (m, genome) was performed on the assembled genome using the euglenozoa_odb10 ortholog set (*n* = 130). A total of 126 complete benchmarking universal single-copy orthologs (BUSCOs) (96.9%) and 4 fragmented BUSCOs (3.1%) were identified from the 130 searched BUSCO groups.

### Data availability.

The sequence employed for the DTU typing was deposited in GenBank under accession number MT231530. The T. cruzi B. M. López assembled genome was deposited in GenBank under accession number WWPY00000000, and raw reads were deposited in the SRA under accession numbers SRR11234856, SRR11234857, and SRR11234858; the BioProject number is PRJNA595079.
